# Detecting Apoptotic Human Lens Epithelial Cells With Transmission Electron Microscopy

**DOI:** 10.7759/cureus.45916

**Published:** 2023-09-25

**Authors:** Konstantina N Sorkou, Paschalis Theotokis, Theodora- Eleftheria Deftereou, Lambropoulou Maria, Soultana Meditskou, Maria Eleni Manthou

**Affiliations:** 1 Department of Ophthalmology, Frimley Park Hospital, National Health Service (NHS), Camberley, GBR; 2 2nd Department of Ophthalmology, School of Medicine, Faculty of Health Sciences, Aristotle University of Thessaloniki, Thessaloniki, GRC; 3 Laboratory of Histology and Embryology, School of Medicine, Faculty of Health Sciences, Aristotle University of Thessaloniki, Thessaloniki, GRC; 4 Laboratory of Experimental Neurology and Neuroimmunology, 2nd Department of Neurology, AHEPA University Hospital, Thessaloniki, GRC; 5 Laboratory of Histology and Embryology, Democritus University of Thrace, Alexandroupolis, GRC

**Keywords:** lens epithelial cells, anterior lens capsule, apoptosis, senile cataract, transmission electron microscopy

## Abstract

Introduction

Cataract formation is a prevalent issue worldwide, and understanding the cellular processes involved is crucial to advancing treatment options. The scope of the study was to explore the presence of apoptotic cells in the lens epithelium of Greek patients with senile cataracts using transmission electron microscopy (TEM).

Methods

Twenty-one patients with senile cataracts were included in this cross-sectional study, and their anterior lens capsules were thoroughly examined. The presence of apoptosis was ultrastructurally investigated, and its association with age, gender, biomicroscopic type of cataract, the coexistence of exfoliation syndrome (XFS), diabetes mellitus, and glaucoma was statistically correlated.

Results

We detected apoptotic cells in nine of the 21 patients. Morphological features indicative of apoptosis in the nuclei included degradation, nuclear membrane irregularity, reduction of nuclear volume, condensation, and margination of chromatin. The cytoplasm either appeared denser or contained vacuoles. Budding with membrane blebbing and pinopode-like projections were frequently observed. Apoptotic cells appeared smaller, exhibiting loose connections with neighboring cells and the basement membrane (BM). Interestingly, apoptotic bodies were also detected.

Conclusions

None of the examined risk factors showed a connection to apoptosis, whereas neighboring lens epithelial cells (LECs) phagocytose apoptotic bodies, seemingly assumed the role of macrophages. Comparing apoptosis rates between populations with different sun exposure levels could help reveal the relationship between ultraviolet B radiation exposure, apoptosis, and cataract formation.

## Introduction

Apoptosis, a genetically controlled process, is characterized as programmed cell death and plays a crucial role in normal cell turnover, embryonic development, and proper immune system functioning [1]. A certain level of apoptosis is necessary for normal function and the maintenance of tissue integrity [1]. Besides physiological pathways, apoptosis often occurs in response to toxic or chemical agents, such as irradiation or drugs, leading to various pathologies, including cancer and neurodegenerative diseases [2]. The capacity to modulate a cell’s life or death offers immense therapeutic potential [2], making apoptosis a widely researched cell death mechanism.

Under normal conditions, apoptosis is typically a rare event [3]. It is estimated that in vivo, the process is remarkably rapid, lasting from two to 24 hours [2,3]. Consequently, only a few cells undergoing apoptosis are present at a single time point. Transmission electron microscopy (TEM) is considered the gold standard method for confirming apoptosis [2], as it provides images of specific morphological features, such as nuclear fragmentation, apoptotic bodies, blebbing, and cytoplasmic or nuclear condensation, which characterize an apoptotic cell [2,4]. However, the combination of (a) the limited number of cells that TEM can feasibly study, (b) the brief duration of apoptosis, and (c) the potential for apoptosis to occur in specific sites within a tissue may result in underestimating apoptosis or producing false-negative results [2,3]. Identifying even a single apoptotic cell by electron microscopy can be challenging [3].

The etiopathogenesis of cataracts has been linked to the apoptosis of lens epithelial cells (LECs) to varying degrees [5-8]. Additionally, recent studies have connected cataract formation and apoptosis to exfoliation syndrome (XFS) [9]. Although TEM is considered the ideal method for confirming apoptosis, studies focusing on LEC apoptosis in cataracts primarily employ immunohistochemical and biochemical techniques [7-9].

This study is part of an experiment where we examined anterior lens capsules (aLCs) from patients with age-related cataracts and exfoliation syndrome (XFS) using TEM for the first time in a Mediterranean population. We have already reported novel ultrastructural abnormalities in XFS patients in the subepithelial region of the aLCs [10,11] and within the basement membrane [12]. The present study aimed to document apoptosis, which is rarely described in TEM studies usually performed on animals. The XFS and non-XFS cataractic groups were evaluated, comparing the presence of apoptosis between the two groups in human LECs and evaluating potential risk factors related to the etiopathology of apoptosis.

## Materials and methods

An observational, cross-sectional study was conducted to investigate the presence of apoptotic cells in the lens epithelium of Greek patients with senile cataracts using TEM. Twenty-one patients, all older than 60 years, with senile cataracts, participated in the study, and 11 also had XFS. Informed consent was obtained from all patients before surgery. The research adhered to the tenets of the Declaration of Helsinki and received approval from the Ethical Committee and the Hellenic Data Protection Authority (Approval Number: ΓΝ/ΕΞ/1445-1/27.04.2015).

The aLC from each patient was collected during an uneventful phacoemulsification performed by the same surgeon. A 5-5.5 mm circular portion of the central aLC was carefully removed using continuous curvilinear capsulorrhexis with forceps and was immediately fixed in a neutral-buffered 3% glutaraldehyde solution for 90 minutes in the operating room.

All specimens were post-fixed in 2% osmium tetroxide (OsO4) and transferred to the Laboratory of Histology and Embryology of the AHEPA University Hospital, Thessaloniki, Greece. Subsequently, all capsules were dehydrated at increasing ethanol concentrations and embedded in Epon 812. Semi-thin sections (1-3 μm) were obtained from the center, perpendicular to the capsule plane, and were stained with 1% cyanetoluidine for analysis with light microscopy. Ultrathin golden sections (60-80 nm) were stained with uranyl acetate and lead citrate and examined using a JEOL JEM-1011 transmission electron microscope (JEOL Ltd., Tokyo, Japan), operating at 80 kV. Six different areas of the aLCs were examined, with at least 25 cells in each case.

Statistical analysis of apoptosis was performed in association with the following factors: age, gender, biomicroscopic type of cataract, and coexistence of exfoliation syndrome, diabetes mellitus, and open-angle glaucoma, either primary or secondary related to XF syndrome. Data were analyzed using IBM SPSS software for Windows, Version 23.0 (Armonk, NY: IBM Corp.). The Shapiro-Wilk-Wilk was used to assess normality in the distribution. Student’s t-test was used for quantitative data and the Chi-square test or Fisher’s exact test for nominal data, depending on the expected cell counts. The statistical significance cutoff was p<0.05.

## Results

The demographic data of the patients included in the study are presented in Table 1.

**Table 1:** Demographic and statistical data of the study participants

Variable	N (%)
Gender	
Male	7 (33.3%)
Female	14 (66.7%)
Biomicroscopic type of cataract	
Cortical	9 (42.9%)
Nuclear	10 (47.6%)
Posterior subcapsular	2 (9.5%)
Other pathologies	
Exfoliation syndrome (XFS)	11 (52.4%)
Diabetes mellitus	6 (28.6%)
Glaucoma	3 (14.3%)
Apoptotic lens epithelial cells (LECs)	9 (42.9%)

The study population had a mean age of 72.2 ± 7.9 years. Apoptosis was identified in one or two cells in nine patients out of approximately 25 observed cells per sample. Apoptotic cells were present in six out of 11 patients with XFS and three out of 10 without XFS (p=0.575; Table 2).

**Table 2:** Statistical analysis between patients with and without apoptotic LECs IOP: intraocular pressure; LECs: lens epithelial cells; SD: standard deviation

Variable	Apoptotic LECs	No apoptotic LECs	p-value
Age (years)	70.77 (SD 7.66)	73.41 (SD 8.36)	0.468
IOP (mmHg)	15.67 (SD 4.24)	14.67 (SD 3.08)	0.538
Exfoliation syndrome	6	5	0.575
Gender			
Male	4	3	0.642
Female	5	9	
Biomicroscopic type of cataract			
Cortical	3	6	0.368
Nuclear	5	5	
Posterior subcapsular	1	1	
Other pathologies			
Glaucoma	2	1	0.388
Diabetes mellitus	3	3	0.523

**Figure 1:**
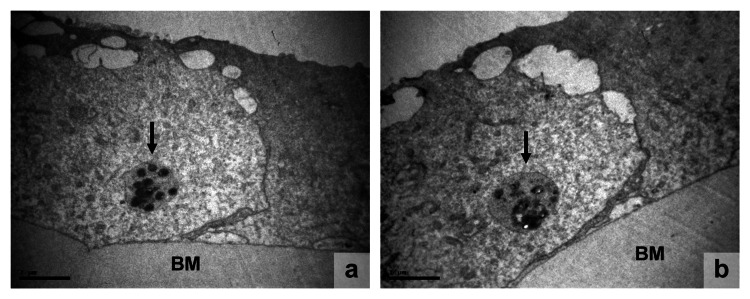
LECs micrographs of different specimens, (a) x 14000 and (b) x 12000 (a, b) Apoptotic bodies, being phagocyted, are indicated with arrows inside cells. The cytoplasms of LECs appear with low density and incipient vacuolation. Intercellular vacuoles are seen at the contact site, between adjacent cells. LECs: lens epithelial cells; BM: basement membrane

**Figure 2:**
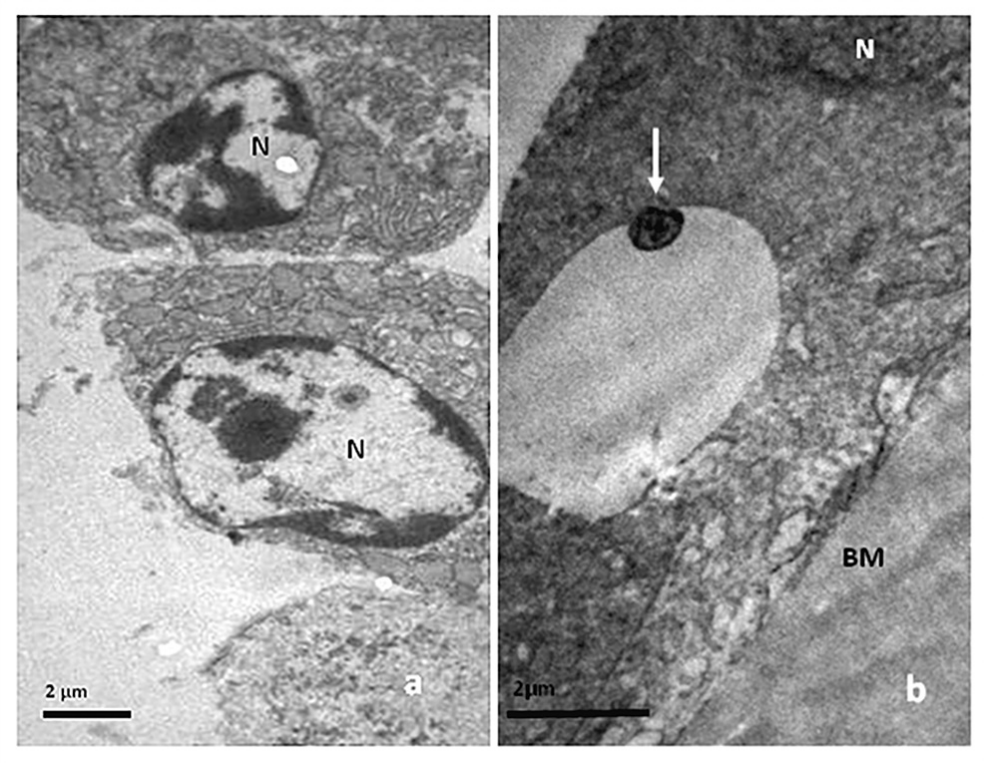
LECs micrographs of different specimens, (a) x 6000 and (b) x 10000 (a) Degradation of nuclei and margination of chromatin. The process of budding has just begun. Intercellular space has widely broadened, and contact between cells seems to be lost. (b) A small, round, electron-dense apoptotic body (arrow) is present within an empty vacuole of the cell. In the upper right corner, part of the cell’s nucleus (N) is observed. LECs: lens epithelial cells; N: nucleus; BM: basement membrane

**Figure 3:**
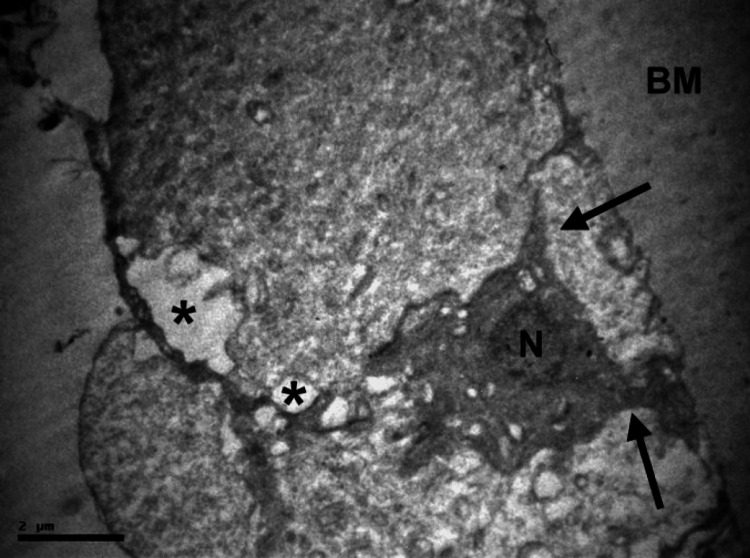
LECs micrograph of another specimen, x 10000 A smaller cell in the middle with pinopode-like structures (black arrows), still in contact with neighboring cells. The cell appears shrunk, with dense cytoplasm and nucleus (N), while budding has started. Intracellular cytoplasmic vacuoles are seen (asterisks). LECs: lens epithelial cells; N: nucleus; BM: basement membrane

Apoptotic cells were recognized at different stages of the apoptotic process (Figures 1-3).

Degradation of nuclei was evident, characterized by a reduction in volume and often the presence of highly irregular nuclear membranes. Nuclei sometimes appeared uniformly dense but usually exhibited margination of condensed chromatin, with the largest part of the nucleus appearing empty (Figures 2a, 3).

Apoptotic cells typically appeared smaller in size (Figure 3). The cytoplasm was sometimes dense, but it contained numerous cytoplasmic vacuoles in most cases. A reduction in mitochondria and an expansion of the rough endoplasmic reticulum were frequently observed. Budding, along with cytoplasmic membrane blebbing, was a common finding. Many apoptotic cells exhibited pinopode-like projections (Figure 3) and had loose or no connections with the basement membrane and neighboring cells (Figure 2a). When cell-to-cell adhesions were lost, apoptotic cells separated from neighboring cells or created an empty space within the epithelial lining (Figure 2a). In a few cases, apoptotic bodies within phagosomes were observed (Figures 1, 2b). Neighboring LECs, seemingly acting as macrophages, phagocytosed apoptotic bodies. In addition to apoptosis, degenerative lesions were observed, such as diffuse intracellular and extracellular edema, to varying degrees.

## Discussion

Transmission electron microscopy is considered the most specialized method for identifying apoptosis, which is ultrastructurally characterized by distinct morphological changes in the dying cell [2]. Early apoptotic changes include degradation of the nucleus, nuclear membrane irregularity, pyknosis, condensation, and margination of chromatin, and sometimes uniformly dense nuclei [13]. The cell appears shrunken, and the cytoplasm is dense. The budding process follows, characterized by cell membrane blebbing, pinopodes, and cytoplasmic vacuoles. Karyorrhexis is evident at the end of the apoptotic process, and nuclear and cytoplasmic apoptotic bodies form [2]. These bodies result from cell fragmentation and consist of cytoplasm with tightly packed, intact, well-preserved organelles, with or without a nuclear fragment. Membrane-bound apoptotic bodies express potent triggers for phagocytosis on their surfaces. No cellular constituents are released into the surroundings, and no anti-inflammatory cytokines are produced. Macrophages, parenchymal cells, or surrounding cells phagocytose apoptotic bodies, which are then degraded within phagosomes, preventing secondary necrosis [2].

Apoptosis in cataract lenses has primarily been studied ultrastructurally in animal studies and through immunohistochemical and biochemical techniques in humans [4,5,14-17]. These studies have led to conflicting theories concerning the relationship between apoptosis and cataract formation. Some researchers suggested an etiopathological connection between apoptosis and cataracts [5,6], while others opposed this notion [7,8]. Human cataract has been studied in humans ultrastructurally rather recently, with usually no specific description of apoptosis [18-20]. The development of presenile and age-related cataracts is most commonly attributed to ultrastructural pathological changes in the anterior lens epithelial cells [20], although fluid accumulation and electrolyte imbalance in the lens by some are considered to be attributed more to cataract formation than do changes in the lens capsule [18]. In a very recent ultrastructural, non-cataractic study, autophagy was observed with immunofluorescence; nevertheless, no apoptosis was described in LECs [21]. On the other hand, the concomitance of increased autophagy activity and apoptosis in the same LECs from senile cataract patients was described in another study [22], leading to the conclusion that autophagy facilitates cell apoptosis.

Interestingly, it is reported that the incidence of apoptosis is increased in patients with diabetes mellitus [23] and in patients with anterior polar cataracts, making it higher than in age-related cataracts [4]. Our study demonstrates apoptosis in human cataractic lens epithelium using TEM and examines its correlation with age, gender, biomicroscopic type of cataract, XFS, diabetes mellitus, and glaucoma, but no association was established (Table 1).

In a recent terminal deoxynucleotidyl transferase dUTP nick end labeling (TUNEL)-based study, apoptotic rates appeared significantly higher in patients with XFS than those without [9]. Our study found nearly twice as many apoptotic cases with XFS, but the result was not statistically significant (p=0.575). Apoptosis in LECs has also been correlated with other lens pathologies and conditions [17,24-26]. However, none of these conditions were present in our patients’ medical histories.

We must consider the potential influence of sunlight exposure on our Mediterranean population. High-intensity sunlight, a type of ultraviolet B (UVB) radiation, has been linked to senile cataracts in several epidemiological studies [27-29]. The World Health Organization documents higher UVB radiation exposure in Greece compared to northern countries [30]. Since UVB radiation has been connected with both LEC apoptosis [5,6] and senile cataracts [27-29], we speculate that apoptosis could be etiopathologically linked with age-related cataracts through UVB radiation exposure.

Apoptosis of LECs is the important cellular basis of senile cataracts, which results from prolonged exposure to oxidative stress [22]. Could this stress be UVB?

Finding even a single apoptotic cell using TEM can be challenging [3]. Due to the restricted number of cells studied and the limited duration of apoptosis, TEM is known to underestimate the presence of apoptosis [2,3]. It is possible that more patients had apoptotic cells that were not detected at the particular time point or capsule area examined. Therefore, our results represent true positive results, and the incidence of apoptosis may be higher. Although we observed human apoptotic cells in many patients in our randomized sample, TEM is unsuitable for quantitative analysis, and our study cannot provide specific information about the apoptosis rate.

Our study had several important limitations. First, the small sample size of 21 patients restricts the generalizability of the findings and reduces the statistical power to detect significant associations. Second, the study focuses on a Mediterranean population, which may not represent other populations with different sunlight exposures and genetic backgrounds. Third, we used TEM to detect apoptotic cells; however, this method is unsuitable for quantitative analysis as it only examines a restricted number of LECs. Consequently, the study could not provide specific information about the apoptosis rate. The study's cross-sectional design allows for the identification of associations between variables but cannot establish causality or directionality between apoptosis and the risk factors examined. Finally, the study did not account for potential confounding factors, such as the duration and intensity of sunlight exposure or other environmental factors that could potentially influence the apoptotic process and cataract formation.

## Conclusions

In this TEM study, we observed apoptosis in the human lens epithelium of patients with senile cataracts. We found that neighboring LECs phagocytose apoptotic bodies, seemingly assuming the role of macrophages. Although we did not identify a correlation between apoptosis and XFS or other examined risk factors, and we could not quantitatively estimate the apoptosis rate, our findings may be influenced by the increased sunlight and consequent UVB radiation exposure experienced by our Mediterranean population. Comparing apoptosis rates between populations with varying levels of sun exposure could help elucidate the interrelationship between UVB radiation, apoptosis, and cataract formation.
